# A Mathematical Method to Adjust MLC Leaf End Position for Accurate Dose Calculation in Carbon Ion Beam Radiation Therapy Treatment Planning System

**DOI:** 10.1155/2021/6762724

**Published:** 2021-10-21

**Authors:** Yan-Shan Zhang, Yan-Cheng Ye, Jia-Ming Wu

**Affiliations:** ^1^Heavy Ion Center of Wuwei Cancer Hospital, Gansu Wuwei Academy of Medical Sciences, Gansu Wuwei Tumor Hospital, Wuwei City, Gansu Province, China; ^2^Department of Medical Physics, Chengde Medical University, Chengde City, Hebei Province, China; ^3^Department of Radiation Oncology, Yee Zen General Hospital, Taoyuan City, Taiwan

## Abstract

**Introduction:**

We present a mathematical method to adjust the leaf end position for dose calculation correction in the carbon ion radiation therapy treatment planning system.

**Methods and Materials:**

A straggling range algorism of 400 MeV/n carbon ion beam in nine different multileaf collimator (MLC) materials was conducted to calculate the dose 50% point to derive the offset corrections in the carbon ion treatment planning system (ciPlan). The visualized light field edge position in the treatment planning system is denoted as *X*_tang.p_, and MLC position (*X*_mlc.p_) is defined as the source to leaf end midpoint projection on axis for monitor unit calculation. The virtual source position of energy at 400 MeV/n and straggling range in MLC at different field sizes were used to calculate the dose 50% position on axis. On-axis MLC offset (correction) could then be obtained from the position corresponding to 50% of the central axis dose minus the *X*_mlc.p_.

**Results:**

The exact MLC position in the carbon ion treatment planning system can be used as an offset to do the correction. The offset correction of pure tungsten is the smallest among the others due to its shortest straggling range of carbon ion beam in MLC. The positions of 50% dose of all MLC materials are always located in between *X*_tang.p_ and *X*_mlc.p_ under the largest field of 12 cm by 12 cm.

**Conclusions:**

MLC offset should be adjusted carefully at different field sizes in the treatment planning systems especially of its small penumbra characteristic in the carbon ion beam. It is necessary to find out the dose 50% position for adjusting MLC leaf edge on-axis location in the treatment planning system to reduce dose calculation error.

## 1. Introduction

In most commercial photon radiation therapy facilities such as linear accelerators, multileaf collimator (MLC) systems are used to improve the dose profile of the geometry penumbra and the transmission penumbra [1]. MLC was not only used commonly as treatment accessories in photon but also adopted in heavy charged particle therapy such as carbon ion beam treatment [2]. The coincidence between the 50% dose position and the light field of the photon beam cannot be taken for granted with the nondivergent geometry that is found in the curved-leaf linear type of collimator system; the 50% dose position must be verified during MLC system acceptance [3]. Not like the photon, MLC systems utilize designs with rounded leaf ends to improve the coincidence of the radiation 50% point with projected light field edge; the shape of MLC leaf end in the charged particle is rectangular [4]. The straggling range of carbon ion beam in MLC saves troubles with the rounded leaf end design caused by photon attenuation in MLC [5]. One of the most important principles of MLC design is to reduce the differences between the dose 50% points and the projected light field edge on axis [6]. The characteristic of the projected light field edge locations and the definition of MLC position as well as the dose 50% points in the photon treatment planning system need to be corrected before patients' treatment monitor units are calculated. The MLC position in planning and its relative radiation dose 50% point of the carbon ion MLC are also needed to be corrected and implemented in the computerized treatment planning system for accuracy monitor unit calculation [7]. In this work, we illustrate the specific issues to carry out dose calculation of a rectangular end MLC system with an offset correction in a carbon ion beam.

## 2. Materials and Methods

This work presented here was performed with 400 MeV/n on a carbon ion therapy facility established by the Institute of Modern Physics (IMP), China. The IMP affiliated with the Chinese Academy of Sciences (CAS) was founded in 1957 in Lanzhou, China. To take the advantage of full usage of the research facilities at IMP, the National Laboratory of Heavy Ion Accelerator, Lanzhou (NLHIAL), was established at IMP in 1991 [8]. Our Wuwei Heavy Ion Center, Wuwei Cancer Hospital, Gansu, China (WHICH), heavy-ion facility established by IMP, CAS at 2014, was the first-generation commercialized product transformed from a laboratory-based cancer treatment facility in China. Our facility was a modification from the prototype of the Heavy Ion Research Facility in Lanzhou (HIRFL) and started to treat patients in earlier 2020. Our WHICH consists of an ECR ion source, an injection cyclotron SFC (energy constant *K* = 69), and a cyclotron SSC (energy constant *K* = 450) as an injector offering charged particles to the main synchrotron ring to accelerate sufficient particle energy and flux for four treatment rooms' use—room 1, horizontal nozzle alone with scanning beam; room 2 (for clinical use only), vertical+horizontal nozzles of passive scatter beam; room 3, vertical nozzle alone with scanning beam; and room 4, 45° nozzle alone with passive scatter beam for cancer patient treatment. At WHICH, our homemade ciPlan treatment planning system was used for carbon ion dose calculations. Room 1, 3, and 4 are not ready for clinical services. Dose profiles of MLC fields were measured for room 2 passive scatter beam and implemented to the ciPlan for dose calculation in this study.

**Figure 1:**
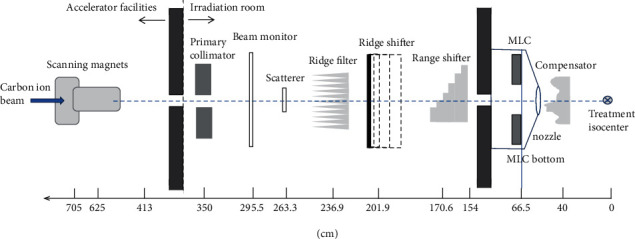
The physical dimensions of carbon ion facility at WHICH.

Nine MLC materials including the straggling range listed at Table 1 were adopted for this study. The subscript denotes the percentage compositions of each MLC material. According to IMP previous Monte Carlo simulation, the platform was v8.2/GEANT4-10-05-patch-01 with QGSP_BERT_HP_EMY package of Gate (GEANT4 Application for Tomographic Emission) [9]. The geometric dimensions of WHICH are showed in Figure 1. All on-axis profiles were measured with a certain visual light field (nominal light field) at a SAD of 263.3 cm to determine the point receiving 50% of the central axis dose. The projection of the nominal light field at SAD 263.3 cm was adopted as a setup condition for dose profile measurements in water phantom, but the geometry of the tangential interaction on the *x*-axis (*X*_tang,p_) was derived from *X*_mlc,p_ (planning system defined leaf position) in ciPlan treatment planning system; furthermore, the corresponding dose 50% point to the central axis dose of *X*_mlc,p_ was calculated by mathematical methods in this study. Once the dose 50% point was decided, the on-axis correction “offset” could be obtained by subtraction of the point corresponding to 50% of the central axis dose from the position of *X*_mlc.p_.

### 2.1. Geometry Specifications

#### 2.1.1. Nominal Light Field

Nominal light field means the size of the visualized light field that is set for patient treatment and for dose profile measurements.

#### 2.1.2. *X*_tang.p_: The Position of Light Field Projection Edge Interaction on Axis

**Figure 2:**
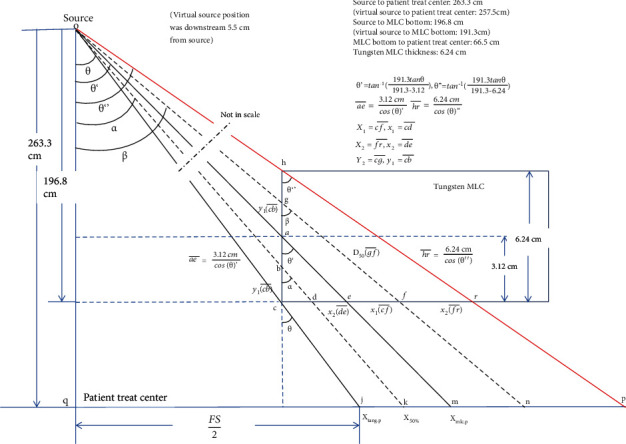
The definition of MLC nominal light field (visualized light field) edge, *X*_tang.p_, the intersection of a line from the source to the leaf tip with an angle of *θ*′, *X*_mlc.p_, and the dose 50% position *X*_50%_ (straggling range) of tungsten in the treatment planning system.

According to Figure 2, the bottom of carbon ion MLC rectangular leaf end determines *X*_tang.p_, which is the intersection of a prolonged line from the source to point *j* with the isocenter horizontal axis at a SAD of 263.3 cm. *X*_tang.p_ is used quantitatively to describe the leaf edge in the treatment planning system, while the nominal light field (visualized light field) edge is used qualitatively by humans to check the boundary of the treatment area.

#### 2.1.3. *X*_mlc.p_: Definition of Leaf Position in Treatment Planning

*X*_mlc.p_ is the intersection of a line from the source to the leaf tip (*m* in Figure 2) with SAD 263.3 cm on the axis. Patient dose calculations are based on this point in the treatment planning system.

#### 2.1.4. The Direction of the MLC

When the MLC travels away from the central axis (the field size becomes larger), the direction is denoted as positive (“+” in all figures). When the MLC travels closer to or crosses over the central axis, the direction is denoted as negative (“–” in all figures).

#### 2.1.5. Virtual Source Position of 400 MeV/n Carbon Ion Beam

A pencil carbon ion beam is spread into a broader beam after passing through the primary collimator, beam monitor, scatterer, ridge filter, ridge shifter, and the range shifter that appears to diverge from a point—this point is so-called the virtual source. The virtual source position may be defined as an intersection point of the back-projection along with the most probable directions of carbon ion motion at the patient surface. Field size magnification of the 50% width of the beam profiles on GAF chromic film with different distances was used for determining the virtual source position of a carbon ion beam.

The virtual source position *f* was measured by the definition below:

(1) $$\begin{array}{c} \frac{FS_{\text{SAD},f}}{{\text{FS}}_{f+g}}=\frac{f}{f+g},\\ f=\frac{g}{\left({\text{FS}}_{f+g}/\text{F}{\text{S}}_{\text{SAD},f}\;\right)-1}, \end{array}$$

where FS_SAD,*f*_ denotes the field size at SAD 263.3 cm. The maximum field size of our WHICH carbon ion beams is 12 cm × 12 cm at the isocenter of 263.3 cm. A field size of 8 cm × 8 cm with gaps upstream or downstream was adopted for the virtual source position measurement in this study.

FS_*f*+*g*_ denotes the field size at SAD 263.3 cm with gaps upstream or downstream; here, we adopted the upstream and downstream with a gap of -15 cm (close to the source) and +15 cm (away from the source), respectively.

*f* is the virtual source position and is the intersection point of the back-projection along with the most probable directions of carbon ion motion at measurement device surface.

#### 2.1.6. GAF Chromic Film for Measuring the Virtual Source Position of 400 MeV/n Carbon Ion Beam

We used GAF chromic EBT3 films (Ashland Specialty Ingredients GP, NJ USA; Lot # 04022001, Exp. Date: April 2021) for determining the virtual source position of 400 MeV/n carbon ion beam in this study. The film processing and dose profile measurements followed the international protocols [10]. A preexposure technique was used for the calibration curve derivation [11]. This was performed by giving each film a priming dose of 2 Gy to homogenize the film density using WHICH facility with a dose of 1 Gy at a carbon ion energy of 400 MeV/u. We then measured the dose homogeneity using a densitometer. Graded doses of 5, 10, 15, 40, 60, 80, 100, 150, and 200 cGy were given to the GAF chromic film to obtain the Hurter-Driffield calibration curve (H-D curve).

All exposed films of depth dose curve were then scanned with an Epson Expression 11000XL scanner in the 48-bit RGB mode (16 bits per color), and the data were saved as tagged image file format (TIFF) and analyzed by the VeriSoft imaging procession software. A red filter was placed on top of the GAF films before scanning to increase the slope of the H-D curve, thereby raising the resolution of the dose-OD curves [12].

The field size derived from dose 50% of the dose profile at isocenter was then compared to upstream and downstream films with a gap of -15 cm and +15 cm for determining the virtual source position.

#### 2.1.7. The 50% Dose Position: *X*_50%_

The radiation field size is defined as the lateral distance between the 50% isodose line (*X*_50%_) at a reference depth. In photon beam, the dose 50% of the central axis dose is determined by the attenuation of radiation in MLC, while in the carbon ion beam, the straggling range dominates the position of *X*_50%_. When the MLC moves near to or away from the central axis (Figure 2), the *X*_50%_ position might locate at point *n* (right to *X*_mlc.p_) or point *k* (left to *X*_mlc.p_), respectively. This depends on the straggling range in MLC (denoted as $\bar{gf}$ or $\bar{bd}$ in Figure 2).

#### 2.1.8. Determination of Dose 50% by Straggling Range of Carbon Ion Beam in MLC

The number of beam nuclei that survive passage through the MLC, *N*, can be determined from the total number of carbon ion particle interaction events in MLC. This particle number is compared with the total number of incident nuclei, *N*_*B*_, as determined from the total number of events in the collision history [13].

(2) $$\begin{array}{c} N=N_{B}e^{-xt/\lambda t}, \end{array}$$

where *x*_*t*_ is the thickness of tungsten and *λt* is the interaction mean free path (MFP), in other words, straggling range in tungsten MLC. Let

(3) $$\begin{array}{c} N=N_{B}e^{-xt/\lambda t}.\\ N=0.5. \end{array}$$

*N*_*B*_ = 1; then, 0.5 = *e*^−*xt*/*λt*^, *x*_*t*_ is the half value layer of a certain carbon ion energy in MLC material.

For example, *λt* = 27 mm for tungsten at a carbon ion energy of 400 MeV/n; then,

(4) $$\begin{array}{c} \ln\left(0.5\right)=\frac{-d50}{27\text{ mm}}. \end{array}$$

*d*_50_ = 1.8711 cm, which means the path length to reduce dose to 50% of a carbon energy 400 MeV/n in tungsten MLC is 1.8711 cm.

Figure 2 shows a schematic drawing of a mathematical model for deriving the on-axis 50% dose position (*X*_50_), *X*_tang,p_ and *X*_mlc,p_ at a SAD of 263.3 cm. In Figure 2, the precise position of the light field edge (*X*_tang,p_) was transformed from *X*_mlc,p_ (denoted as “*m*” in this figure) which was defined as MLC position in the treatment planning system.

Once the MLC position is confirmed, the dose 50% position can be derived by the procedure in the appendix.

#### 2.1.9. Offset Definition

The patient treatment monitor unit calculation was based on *X*_mlc,p_ in the treatment planning system. The definition of the adjustment offset is as follows: The offset is equal to the 50% dose position minus the position of *X*_mlc,p_.

## 3. Results

### 3.1. Virtual Source Position of 400 MeV/n Carbon Ion Beam

The result of virtual source point by field size magnification on films obtained by the back-projection of the 50% width of the beam profiles at different distances was found to be 5.5 cm downstream from the scatterer position in Figure 1. In other words, the virtual source position was 257.8 cm from the patient treatment isocenter and the distance from the virtual source to the bottom of the MLC is 191.3 cm.

### 3.2. On-Axis Offset Correction of Tungsten MLC Leaf End Position in the Treatment Planning System

Patient treatment field size is determined by plan designer according to the lesions of a PTV in the treatment planning system. *X*_tang.p_ is used quantitatively to describe the visualized light field leaf edge, while *X*_mlc.p_ is the intersection of a line from the source to the leaf tip with an angle of *θ* and *θ*′ in the treatment planning system, respectively. The dose 50% position *X*_50%_ (straggling range in MLC) of tungsten was derived by the angle *α* listed in Table 2 once *X*_tang.p_ and *X*_mlc.p_ are determined. The offset corrections listed in Table 2 are equal to the dose of 50% position (*X*_50%_) minus the position of *X*_mlc,p_ (MLC plan position in planning system). Light-radiation agreement and the penumbra defined as dose profile between 20% and 80% are also listed in Table 2.

### 3.3. Secondary Radiation Equivalent Dose and Offset Correction of Different MLC Materials

The results of offset correction calculated by the procedures described in the appendix of nine different MLC materials with different field sizes are listed in Table 3. The largest and smallest offset corrections at the largest field size were pure alumina and pure tungsten, respectively. The secondary radiation equivalent dose (mainly composed of prompt gamma-ray and neutrons in 10^−^4 Sv) simulated by IMP Monte Carlo simulation of the interactions of 400 MeV/n carbon ion beam with nine different MLC materials is also listed in Table 3.

**Figure 3:**
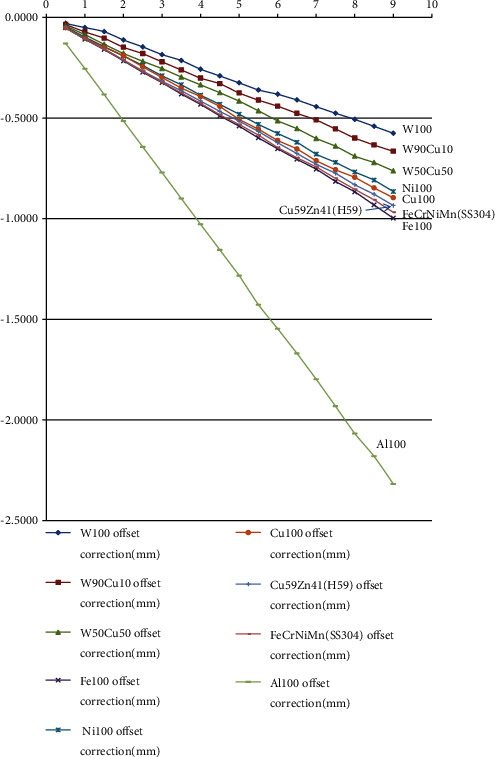
The schematic demonstrates the offset corrections of different materials for MLC. The offset correction is increased because the composition of different metals increased leading to the increment of straggling ranges.

Figure 3 is the schematic demonstration of the nine different MLC material offset corrections. Pure alumina is segregated by the others due to its low *z* characteristic.

## 4. Discussion

The straggling range, as well as the thickness of MLC, was increased when the percentage of copper compositions of tungsten is increased in Table 1. From the weights and mechanical driven point of view, the optimal material of MLC is pure tungsten.

The virtual source position was derived by field size magnification on films obtained by the back-projection of the 50% width of the beam profiles at different distances of the in and out direction (penetrate vertically through the paper) instead of up and down direction (parallel to the MLC movement demonstrated on paper) in Figure 1. It was because the uncertainty of field size magnification on films obtained by up and down direction was larger than in and out direction due to the facility MLC movement which is at up and down direction shown in Figure 1.

The on-axis offset (the 50% dose position minus the planned leaf position) is used for accurate monitor unit calculation. Figure 2 shows *X*_tang,p_, *X*_mlc,p_, and the on-axis position receiving 50% of the central axis dose (point *k* or *n*). In photon beams, when MLC leaf travels close to the central axis, owing to gain enough attenuation, the 50% dose position must project outside *X*_mlc,p_ (right to *X*_mlc,p_) on point *n*. As the MLC leaf travels away from the central axis, the 50% dose projection position moves inside *X*_mlc,p_ (left to *X*_mlc,p_) to point *k* for less attenuation in Figure 2. Not like photons, the carbon ion *X*_50%_ is always located in between *X*_tang,p_ and *X*_mlc,p_ regardless of the field size due to the straggling range which is enough for 50% dose attenuation. This offset adjustment can be of importance in clinical situations of split fields to avoid calculating overdosage or underdosage at treatment.

The maximum field size of our institute carbon ion beam is 12 cm × 12 cm, the corresponding offset and light-radiation agreement of half field size of 6 cm in Table 2 were -0.3689 mm and -0.59767 mm, respectively. The minus sign means the *X*_50%_ located in between *X*_tang,p_ and *X*_mlc,p_. For photon beams, the design of rounded leaf end structure reduces the distance of *X*_50%_ to *X*_tang,p_ and *X*_mlc,p_, while in carbon ion beams, the rectangular leaf end has the same effect with rounded leaf end due to the straggling range of heavy charged particle in MLC.

Figure 3 shows the alumina was not suitable for MLC due to its low *z* material. The offset correction was increased because the composition of different metals of all kinds of alloys increased leading to the increment of straggling ranges in MLC.

The difference of secondary radiation equivalent dose of tungsten and alumina was only 1.5 × 10^−4^ Sv showed in Table 3; considering the weights and movement flexibility, tungsten is still the best choice for fabricating MLC.

## 5. Conclusions

In this study, we illustrate that the accumulated and planned radiation doses may not always be in agreement for MLC treatment fields at a carbon ion beam treatment planning system unless the offset is carefully adjusted.

It is necessary to find out the dose 50% position for adjusting MLC leaf edge on-axis location in the treatment planning system to reduce dose calculation error.

We should keep in mind that patient treatment monitor unit calculations at extreme settings such as a split field in carbon ion beam could result in significant uncorrectable underdosage or overdosage in treatment planning calculation.

## Figures and Tables

**Table 1:** Nine MLC materials including the percentage compositions of each MLC materials denoted as subscript symbols with straggling range were listed for this study.

MLC material	Composition	Density (g/cm^3^)	MLC thickness (cm)	Struggling range (cm)
W_100_	100%	19.3	6.24	2.62
W_90_Cu_10_	W-90% Cu-10%	17.34	6.7	2.85
W_50_Cu_50_	W-50% Cu-50%	12.25	8.32	3.66
Fe_100_	100%	7.87	11	5
Ni_100_	100%	8.9	9.6	4.3
Cu	100%	8.96	10	4.5
Cu_59_Zn_41_(H59)	Cu-59% Zn-41%	8.4	10.6	4.8
FeCrNiMn(SS304)	Fe-69.5%, Cr-19, Ni-9.5%Mn-2%	7.92	10.8	4.9
Al_100_	100%	2.7	27	13

**Table 2:** The tungsten MLC leaf end offset correction of 400 MeV/n carbon ion beam at different fields.

Field size/2 (cm)	Θ (degree)	Θ (radian)	*θ*′	*θ* ^″^	*α* (*d*_50_) (radian)	Projection on the *x*-axis of *α* (*d*_50_)	Projection on the *x*-axis of *θ* (light field, cm)	Projection on the *x*-axis of *θ*′ (MLC plan position, mm)	MLC plan position shift to *d*_50_ (mm)	Light-radiation agreement (mm)	Penumbra (20%-80%) (mm)
0.5	0.1088	0.0019	0.0019	0.0020	0.0019	0.5050	0.5	0.5081	-0.0301	-0.0504	1.010
1	0.2176	0.0038	0.0039	0.0039	0.0038	1.0110	1	1.0161	-0.0510	-0.11004	1.160
1.5	0.3264	0.0057	0.0058	0.0059	0.0058	1.5165	1.5	1.5241	-0.0763	-0.16515	1.310
2	0.4352	0.0076	0.0077	0.0078	0.0077	2.0197	2	2.0322	-0.1248	-0.19739	1.460
2.5	0.5440	0.0095	0.0096	0.0098	0.0096	2.5230	2.5	2.5403	-0.1729	-0.22978	1.610
3	0.6528	0.0114	0.0116	0.0118	0.0115	3.0285	3	3.0483	-0.1979	-0.28534	1.760
3.5	0.7616	0.0133	0.0135	0.0137	0.0134	3.5322	3.5	3.5564	-0.2421	-0.32171	1.910
4	0.8704	0.0152	0.0154	0.0157	0.0153	4.0368	4	4.0644	-0.2767	-0.36769	2.060
4.5	0.9791	0.0171	0.0174	0.0176	0.0172	4.5414	4.5	4.5725	-0.3112	-0.41367	2.210
5	1.0879	0.0190	0.0193	0.0196	0.0192	5.0460	5	5.0805	-0.3458	-0.45967	2.360
5.5	1.1967	0.0209	0.0212	0.0216	0.0211	5.5525	5.5	5.5886	-0.3606	-0.52544	2.510
6	1.3054	0.0228	0.0232	0.0235	0.0230	6.0598	6	6.0967	-0.3689	-0.59767	2.660
6.5	1.4142	0.0247	0.0251	0.0255	0.0249	6.5618	6.5	6.6047	-0.4288	-0.61831	2.810
7	1.5229	0.0266	0.0270	0.0274	0.0268	7.0690	7	7.1128	-0.4379	-0.68975	2.960
7.5	1.6316	0.0285	0.0289	0.0294	0.0288	7.5736	7.5	7.6208	-0.4724	-0.73581	3.110
8	1.7403	0.0304	0.0309	0.0314	0.0307	8.0782	8	8.1289	-0.5068	-0.78189	3.260
8.5	1.8490	0.0323	0.0328	0.0333	0.0326	8.5828	8.5	8.6369	-0.5413	-0.82798	3.410
9	1.9577	0.0342	0.0347	0.0353	0.0345	9.0874	9	9.1450	-0.5757	-0.87409	3.610

**Table 3:** The MLC leaf end offset correction and secondary radiation equivalent dose for different materials of 400 MeV/n carbon ion beam at different field sizes.

Field size/2 (cm)	1 cm^2^	2 cm^2^	3 cm^2^	4 cm^2^	5 cm^2^	6 cm^2^	7 cm^2^	8 cm^2^	9 cm^2^	Secondary radiation equivalent dose (10^−4^ Sv)
	Offset correction (mm)									
W_100_	-0.05101	-0.12479	-0.19793	-0.27667	-0.34578	-0.36887	-0.43788	-0.50684	-0.57572	4.046 7
W_90_Cu_10_	-0.07208	-0.14874	-0.2208	-0.30202	-0.36022	-0.44136	-0.49946	-0.58049	-0.64328	3.8475
W_50_Cu_50_	-0.08587	-0.17915	-0.25441	-0.33522	-0.41607	-0.51409	-0.61493	-0.68951	-0.76274	3.613 8
Fe_100_	-0.10829	-0.21594	-0.32435	-0.43254	-0.54067	-0.65289	-0.7421	-0.8669	-0.9768	2.819 4
Ni_100_	-0.09836	-0.1921	-0.29042	-0.3864	-0.48112	-0.57674	-0.67966	-0.76811	-0.86531	2.696 4
Cu_100_	-0.10312	-0.19007	-0.299	-0.39392	-0.50628	-0.61128	-0.71152	-0.79512	-0.89624	2.971 6
Cu_59_Zn_41_(H59)	-0.10505	-0.21154	-0.31712	-0.41582	-0.5155	-0.6313	-0.7299	-0.84279	-0.94332	2.935 2
FeCrNiMn(SS304)	-0.10681	-0.21337	-0.3227	-0.42765	-0.53213	-0.62525	-0.73477	-0.84632	-0.95621	2.823 1
Al_100_	-0.25469	-0.51498	-0.77095	-1.02784	-1.28281	-1.54666	-1.79796	-2.06729	-2.31803	2.553 8

## Data Availability

All experiment data can be provided only per the reviewer's requests.

## Appendix

### A Mathematical Demonstration to the Derivation of the Leaf End Radiation Dose 50% Position of MLC

Once the MLC position is conformed, the dose 50% position can be derived by the procedure according to Figure 2.

*θ*′ = tan^−1^(191.3tan*θ*/191.3 − 3.12) and *θ*^″^ = tan^−1^(191.3 tan*θ*/191.3 − 6.24) (196.8 cm downstream 5.5 cm to 191.3 cm— the virtual source position)

(5) $$\begin{array}{c} \bar{\text{ae}}=\frac{3.12\text{ cm}}{\cos\left(\theta^{\prime }\right)},\bar{\text{hr}}=\frac{6.24\text{ cm}}{\cos\left(\theta^{\prime \prime }\right)},\\ X_{1}=\bar{cf},x_{1}=\bar{cd},\\ X_{2}=\bar{fr},x_{2}=\bar{de},\\ Y_{1}=\bar{cg},y_{1}=\bar{cb}. \end{array}$$

When MLC closes to the central axis, the *D*_50_ penetration trajectory is estimated to locate in the region of $\bar{\text{haer}}$

(6) $$\begin{array}{c} \theta^{\prime \prime }=\tan^{-1}\left(\frac{191.3\;\tan\theta }{191.3-6.24}\right),\\ 6.24\;\tan\theta^{\prime \prime }=X_{1}+X_{2},X_{2}=191.3\tan\theta^{\prime \prime }-191.3\tan\beta ,\\ X_{1}=\bar{cf},x_{1}=\bar{cd},\\ X_{2}=\bar{fr},x_{2}=\bar{ce},\\ Y_{1}=\bar{cg},y_{1}=\bar{cb},\\ x_{1}=\bar{cd},\\ x_{2}=\bar{de},\\ y_{1}=\bar{cb},\\ X_{1}^{2}+Y_{1}^{2}=D_{50}^{2},\\ X_{1}=Y_{1}\tan\beta ,\\ X_{1}^{2}+{\left(\frac{X1}{\tan\beta }\right)}^{2}={\left(D_{50}\right)}^{2},\\ X_{1}^{2}\left(1+\frac{1}{\tan^{2}\;\beta }\right)={\left(D_{50}\right)}^{2},\\ 6.24\tan\theta^{\prime \prime }=X_{1}+191.3\tan\theta^{\prime \prime }-191.3\tan\beta ,\\ X_{1}=191.3\tan\beta -191.3\tan\theta^{\prime \prime }+6.24\tan\theta^{\prime \prime },\\ {\left(191.3\tan\beta -191.3\tan\theta^{\prime \prime }+6.24\;\tan\theta^{\prime \prime }\right)}^{2}\left(1+\frac{1}{\tan^{2}\;\beta }\right)={\left(D_{50}\right)}^{2},\\ \left[\theta^{\prime \prime }=\tan^{-1}\left(\frac{191.3\;\tan\theta }{191.3-6.24}\right)\right]. \end{array}$$

*X*_1_ can be derived by another way below

(7) $$\begin{array}{c} X_{1}=191.3\tan\beta -191.3\tan\theta ,\\ X_{1}=D_{50}\sin\beta ,\\ =D_{50}. \end{array}$$

When MLC is far from central axis, the *d*_50_ penetration trajectory is estimated to locate in region of $\;\bar{\text{ace}}$.

(8) $$\begin{array}{c} =d_{50}. \end{array}$$

[*θ* = tan^−1^(FS/2/(263.3 − 5.5))] (virtual source position was downstream 5.5 cm from source)

(9) $$\begin{array}{c} \theta^{\prime }=\tan^{-1}\left(\frac{191.3\tan\theta }{191.3-3.12}\right). \end{array}$$

*θ* is determined by FS/2; *α* can be added by an increase small angle from *θ* to *θ*′; then, *X*_50%_ is found once *d*_50_ reach MLC straggling range.

The derivation below is another way to find *d*_50_

(10) $$\begin{array}{c} 3.12\;\tan\theta^{\prime }=x_{1}+x_{2},x_{2}=191.3\tan\theta^{\prime }-191.3\tan\alpha ,\\ x_{1}^{2}+y_{1}^{2}=d_{50}^{2},\\ x_{1}=\bar{cd},\\ x_{2}=\bar{de},\\ y_{1}=\bar{cb},\\ x_{1}=y_{1}\tan\alpha ,\\ x_{1}^{2}+{\left(\frac{x1}{\tan\alpha }\right)}^{2}={\left(d_{50}\right)}^{2},\\ x_{1}^{2}\left(1+\frac{1}{\tan^{2}\;\alpha }\right)={\left(d_{50}\right)}^{2},\\ 3.12\tan\theta^{\prime }=x_{1}+191.3\tan\theta^{\prime }-191.3\tan\alpha ,\\ x_{1}=191.3\tan\alpha -191.3\tan\theta^{\prime }+3.12\tan\theta^{\prime },\\ {\left(191.3\tan\alpha -191.3\tan\theta^{\prime }+3.12\;\tan\theta^{\prime }\right)}^{2}\left(1+\frac{1}{\tan^{2}\;\alpha }\right),={\left(d_{50}\right)}^{2},\left[\theta^{\prime }=\tan^{-1}\left(\frac{191.3\tan\theta }{191.3-3.12}\right)\right]. \end{array}$$
